# Occupational history of psychosocial work environment exposures and risk of autoimmune rheumatic diseases – a Danish register-based cohort study

**DOI:** 10.5271/sjweh.4220

**Published:** 2025-05-01

**Authors:** Helena Breth Nielsen, Camilla Sandal Sejbaek, Lene Wohlfahrt Dreyer, Ida EH Madsen, Esben Meulengracht Flachs, Karin Sørig Hougaard

**Affiliations:** 1 The National Research Centre for the Working Environment, Copenhagen, Denmark.; 2 Department of Occupational and Environmental Medicine, Copenhagen University Hospital – Bispebjerg and Frederiksberg Hospital, Copenhagen, Denmark.; 3 Center for Rheumatic Research Aalborg (CERRA), Department of Rheumatology, Aalborg University Hospital, Aalborg University, Aalborg, Denmark.; 4 National Institute of Public Health, University of Southern Denmark, Copenhagen, Denmark.; 5 Department of Public Health, University of Copenhagen, Copenhagen, Denmark.

**Keywords:** autoimmune disease, JEM, job-exposure matrix, rheumatoid arthritis, stressors, systemic lupus erythematosus, systemic sclerosis

## Abstract

**Objectives:**

This population-based cohort study examined the association between psychosocial work environment exposures and autoimmune rheumatic diseases, including rheumatoid arthritis (RA), systemic sclerosis (SS), and systemic lupus erythematosus (SLE).

**Methods:**

The total Danish working population, 19–58 years of age (N=2 319 337) was followed from 1997–2018 (37 529 977 person years). Quantitative demands, decision authority, emotional demands, job insecurity, physical violence, role conflicts and possibilities for development at work, as well as a combined psychosocial index were assessed by job-exposure matrices (JEM) and linked with diagnoses of autoimmune rheumatic diseases, ie, RA, SS, and SLE identified in The Danish National Patient Registry. For each psychosocial work environment exposure, recent exposure, accumulated exposure, and number of years with high exposure level were calculated for every employee. Associations with autoimmune rheumatic diseases were assessed by Poisson regression analyses.

**Results:**

The results show that employees in occupations with higher decision authority and, to some degree, possibilities for development at work, have lower risks of autoimmune rheumatic diseases, while employment in occupations with high risk of physical violence involves a higher risk of rheumatoid arthritis. No association was observed for job insecurity or role conflicts at work. The results on quantitative demands, emotional demands and the psychosocial index were less conclusive.

**Conclusion:**

These findings generally do not support that psychosocial work environment exposures are major risk factors for autoimmune rheumatic diseases, but low decision authority, possibilities for development at work, physical violence and possibly the sum of recent adverse psychosocial exposure may be of importance.

Autoimmune rheumatic diseases, including rheumatoid arthritis (RA), systemic sclerosis (SS) and systemic lupus erythematosus (SLE), constitute a heterogeneous group of diseases (1). Apart from affecting joints and muscles, they are characterized by general nonspecific symptoms, lung, kidney, and neurological involvement and higher morbidity and mortality (1). Not surprisingly, autoimmune rheumatic diseases lower self-reported quality of life (2–4). These diseases are all more prevalent among women than men, are mainly diagnosed around midlife (5–7), and present with increasing time trends in incidence (5–8), possibly due to growing awareness and better diagnostics (8). RA has the highest incidence rate in Denmark (35.5 diagnoses per 100 000 person years) (7) followed by 2.4 in both SS and SLE (5, 6).

The etiology of autoimmune rheumatic diseases is not yet known and few modifiable risk factors are identified. Autoimmune diseases are believed to arise from an interplay between genetic and environmental factors (9). The importance of genetic predisposition is widely recognized in RA, but non-genetic factors is estimated to account for 40–70% of disease risk (10). Obestity and especially smoking are suggested to play roles in disease pathogenesis (9, 11). Psychosocial factors are little studied but are suggested to influence onset of autoimmune rheumatic diseases among genetically predisposed individuals and to enhance disease activity (9, 11). Psychological stress may disrupt the hypothalamic-pituitary-adrenal (HPA) axis and autonomic nervous system function and could lead to autoimmune disease (12, 13). Psychosocial factors could also trigger disease indirectly through change in unhealthy behaviors, such as smoking (9).

There is growing evidence that childhood psychosocial trauma increases the risk of autoimmune disease in adult life (14–16). Among adults, one study observed an association between perceived stress and incident inflammatory arthritis (17), the main criteria in classification of RA (18), among people at risk of developing RA. A few case–control studies also found RA cases to more often report major stressful life-events in the year before disease onset compared with controls (19–21). Finally, a recent study on postmenopausal women reported higher risk of RA and SLE with stressful life events (22). Most studies are, however, limited by small sample sizes and risk of recall and selection bias. Interestingly, several studies also link more extreme types of stress-full events among veterans with autoimmune diseases (23–25). One study of 666 269 veterans found higher relative risk for all examined autoimmune diseases, including RA and SLE, among veterans with post-traumatic stress disorder (PTSD) compared to those without PTSD (23). Work-related common psychosocial stressors have however not gained much attention in this respect. Studies report links between decision latitude (26, 27), psychological job demands (26), job strain (26), conflict at work (28), change of work place (28), increased responsibility at work (28), shift work (29) and development of RA. Yet, all of these studies build on questionnaire data, implicating risks of recall and selection bias, and none assessed longitudinal work histories in large working population. Finally, some occupational stressors, such as physical violence, remain to be studied.

The present study aims to examine the association between psychosocial work environment exposures and RA, SS and SLE. We hypothesize that both recent and accumulated exposure and the number of years with high exposure to psychosocial work stressors increase the risk of autoimmune rheumatic disease.

## Methods

This study follows a pre-specified study protocol (30) with additional information on the methods. Appendix A1 in the supplementary material (www.sjweh.fi/article/4220) describes deviations from the protocol.

In this longitudinal register-based cohort study, we included employees and self-employed from the Danish Occupational Cohort with eXposure data (DOC*X), see flowchart in supplementary appendix A2. DOC*X includes all Danish employees ≥16 years old, gainfully employed for ≥1 year from 1976 onwards (31). Here yearly information on employment status, occupation and demographics and several job-exposure matrices (JEM) are available (31). To obtain the full work history of the study population, it was restricted to gainfully employed individuals born 1 January 1960 to 31 December 1999 (the latter turning 19 years old in 2018); with ≥1 valid DISCO-88 occupational code. Employees immigrating after their 18^th^ birthday were excluded.

We excluded individuals diagnosed with RA, SS, SLE or juvenile idiopathic arthritis prior to their 19^th^ birthday and individuals with prevalent diagnoses before baseline in 1997. The Danish National Patient Register (DNPR) holds information on inpatients at Danish somatic hospitals since 1977 and outpatients since 1995 (32). To exclude prior unregistered autoimmune rheumatic disease, a two-year washout period for also prevalent outpatient contacts was applied (ie, 1995–1996). Hence, the population was followed up from 1 January 1997 or the year an individual had ≥1 year of registered employment and had turned 19 years old, which ever came last. Participants were followed until one of the following occurred: death, emigration, clinical end-point (RA, SS or SLE), or end of follow-up at 31 December 2018, which ever came first.

### Psychosocial work environment exposures

We used JEM on psychosocial work exposures (33, 34), all based on self-reported survey information and constructed with sex- and age-specific exposure values for each occupational code. Quantitative demands, decision authority, emotional demands, job insecurity and physical violence are based on the *Work Environment and Health in Denmark* 2012 survey. Estimates were extrapolated to all previous and future calendar years (33). Role conflicts and possibilities for development at work are based on the *Danish Work Environment Cohort Study* 2000 and 2005 surveys. The 2000 estimates were extrapolated to 1979–2000 and 2005 estimates to 2001–2018 (34). Individual psychosocial work exposures were assigned since 1979 or the year of their 19^th^ birthday. For each exposure, we used age- and sex-specific JEM estimates for each job group at the 4-digit occupational code level. If no JEM estimate was available at the four-digit-level, the 3-digit level was used, and so forth. Further descriptions of the JEMs including occupations with high and low exposure levels can be found in the articles and supplementary appendices of (33–35).

If an individual was employed for one year, but no information on occupation was available, we extrapolated the previous occupation, based on occupations up to five years back in time (7% of observations). If no codes were available for extrapolation or no JEM estimates were available for an occupational code, we assigned the JEM exposure estimate for missing occupational code.

**Figure 1:**
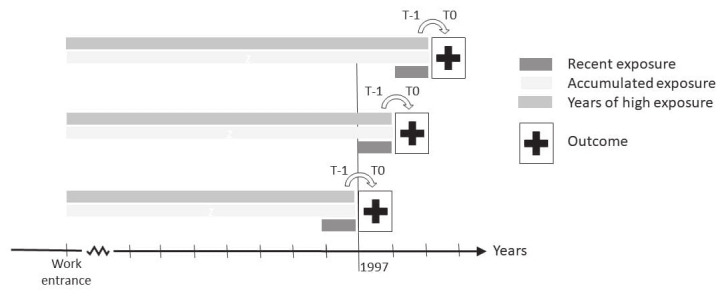
Illustration of the study design. Employees were followed from 1 January 1997 to 31 December 2018 for incident diagnosis of autoimmune rheumatic disease at time (t) zero (t0). This was linked with the occupational history of psychosocial work environment exposures from entrance to the labor market since 1979. The psychosocial work environment exposures were assessed as recent exposure measured in the year before (at t-1); the accumulated exposure, measured from work-entrance and until and including the year before (at t-1); and the number of years with high exposure, measured from work entrance and until and including the year before (at t-1).

Supplementary appendix A3 describes the included psychosocial exposures. Furthermore, a psychosocial index was constructed by summing up the number of adverse psychosocial work environment exposures. Adverse psychosocial work environment exposure levels were defined as high (4^th^ quartile) for quantitative demands, emotional demands, job insecurity, physical violence, and role conflicts at work, and low (1^st^ quartile) for decision authority and possibilities for development at work. Each psychosocial exposure was evaluated as time-varying by (see figure 1): (i) recent exposure: quartile of annual exposure level within the past year [measured at time (t) minus one year (t-1)]; (ii) accumulated exposure: sum of exposure levels from entrance to the labor market and until and including recent exposure, (not calculated for the probability-based JEM measures: physical violence, job insecurity and role conflict); (iii) high exposure years: number of years with an adverse exposure level from entrance to the labor market and until and including the recent exposure.

### Autoimmune rheumatic diseases

We identified incident diagnoses of autoimmune rheumatic diseases as: RA, SS and SLE from in- and outpatient contacts in the DNPR, defined by ICD-8 and ICD-10 codes (table 1). Information includes diagnoses and date of admission and is linked to other registers by the personal Danish identification number (32). We included cases at their second autoimmune rheumatic disease registration (RA, SS and SLE), to reduce misclassification (6, 36). Likewise, the separate outcome analyses included cases at their second registration of RA, SS or SLE, respectively.

**Table 1:** The International Classification of Diseases (ICD) -8 and -10 codes used to identify the included autoimmune rheumatic diseases. ICD codes in parenthesis () are formerly used ICD-10 codes.

Diagnosis	ICD-10 codes	ICD-8 codes
Rheumatoid arthritis	M05, M050, (DM050A), M051, M051A-F, M052, M053, (M053A-E), M058, M059, M06, M060, M062, M064, M068, M069	71219, 71229, 71238, 71239, 71259
Systemic sclerosis	M34, M340, M341, M342, M342A, M342B, M348, M348B, (DM348C), M349	73400, 73401, 73402, 73408, 73409
Systemic lupus erythematosus	M32, M321, (M321A-E), M328, M329	73419

### Covariates

We constructed a directed acyclic graph based on previous literature, see protocol (30), and included the following covariates: age (continuous); sex (women/men); calendar year (by 5-year intervals); highest attained education (primary or lower secondary/upper secondary/short cycle tertiary/bachelor or equivalent/master or higher); ethnicity (Danish/immigrants or descendants); years of non-employment (0, 1–5, 6–10, >10 years); family income (annual quartiles); years of smoking (JEM-based estimates (37), 0, 1–5, 6–10, >10 years in the highest annual quartile of smoking probability); years of obesity (JEM-based estimates (37), 0, 1–5, 6–10, >10 years in the highest annual quartile of body mass index (BMI) probability). Sex and ethnicity were determined at baseline. Other co-variates were time-dependent and resolved annually with a one-year lag (t-1), except age and calendar year, which were set yearly (at t0).

### Statistical analyses

As exposure measures and covariates were updated yearly, we included a 1-year lag time (t-1) in exposure relative to the outcome measure (t0), see figure 1. Associations were analyzed using multilevel Poisson regression analyses, with occupational group (first digit of the Disco-88 code) as level. The logarithm of person-years at risk was used as offset to account for unequal follow-up times and we included a scale parameter to account for overdispersion. We analyzed the association between each psychosocial work exposure and all autoimmune rheumatic diseases (ALL), ie, RA, SS and SLE combined, and in separate analyses for each disease.

Results are presented with incidence rate ratio (IRR) estimates and 95% confidence intervals (CI) from a minimal adjusted model with age and sex; the main model 1 adjusted for: age, sex, calendar year, ethnicity, and highest attained education; and a model 2 (possibly over-adjusted): model 1 plus years of non-employment, family income, smoking and obesity.

### Post hoc sensitivity analyses

We included the following sensitivity analyses to test: (i) possible implications of diagnostic lag time, by testing three- and five-year lag periods between exposure and outcome; (ii) influence of adjustment for physical job strain, due to its close link with occupational group; (iii) full follow-up since 1979, to see if prevalent out-patient cases in 1995 or higher risk of being registered with autoimmune rheumatic disease across the years biased the results; and (iv) exclusion of quantitative job demands from the psychosocial index, as previous studies with this JEM revealed somewhat puzzling findings (33).

## Results

During follow-up (1997–2018), 11 159 diagnoses of the three diseases (in ALL: RA: 9610, SS: 225 and SLE: 1324) were identified in the study population. Table 2 presents demographic characteristics. The mean age was 34.8 (standard deviation 9.6) years, and men (52%), Danish origin (93%) and upper secondary education (46%) contributed more to the populations’ time at risk.

**Table 2:** Background characteristics of the study population at the last year of follow-up (N) and total person years (PY) during follow-up.

		N	%	PY	%
Total		2 319 337	100.00	37 529 977	100.00
Sex					
	Women	1 115 996	48.12	17 999 825	47.96
	Men	1 203 341	51.88	19 530 152	52.04
Age (years old)					
	18–24	319 596	13.78	6 559 895	17.48
	25–34	563 217	24.28	12 288 611	32.74
	35–44	535 348	23.08	11 901 533	31.71
	45–58	901 176	38.85	6 779 938	18.07
Calendar year					
	1997–2003	10 938	0.47	9 218 372	24.56
	2004–2008	10 406	0.45	8 017 337	21.36
	2009–2013	13 016	0.56	9 445 913	25.17
	2014–2018	2 284 977	98.52	10 848 355	28.91
Ethnicity					
	Danish	2 140 025	92.27	35 020 349	93.31
	Immigrant or descendant	119 938	5.17	1 386 842	3.70
	Missing	59 374	2.56	1 122 786	2.99
Highest education level attained					
	Primary or lower secondary	496 150	21.39	10 088 418	26.88
	Upper secondary	1 054 119	45.45	17 429 531	46.44
	Short cycle tertiary	115 411	4.98	1 631 778	4.35
	Bachelor or equivalent	382 184	16.48	4 863 588	12.96
	Master or higher	203 920	8.79	2 209 294	5.89
	Missing	67 553	2.91	1 307 368	3.48
Family income					
	1^st^ quartile	590 274	25.45	9 498 056	25.31
	2^nd^ quartile	553 404	23.86	9 057 705	24.13
	3^rd^ quartile	557 155	24.02	8 939 758	23.82
	4^th^ quartile	555 093	23.93	8 771 147	23.37
	Missing	63 411	2.73	1 263 311	3.37
Smoking (years)					
	0	685 428	29.55	12 753 694	33.98
	1–5	768 515	33.14	12 802 939	34.11
	6–10	385 795	16.63	5 596 128	14.91
	>10	479 599	20.68	6 377 216	16.99
Obesity (years)					
	0	1 402 126	60.45	21 444 959	57.14
	1–5	220 869	9.52	4 535 395	12.08
	6–10	137 788	5.94	3 442 988	9.17
	>10	558 554	24.08	8 106 635	21.60
Non-employment (years)					
	0	857 455	36.97	17 640 451	47.00
	1–5	1 081 017	46.61	15 680 415	41.78
	6–10	206 323	8.90	2 434 072	6.49
	>10	174 542	7.53	1 775 039	4.73

**Figure 2:**
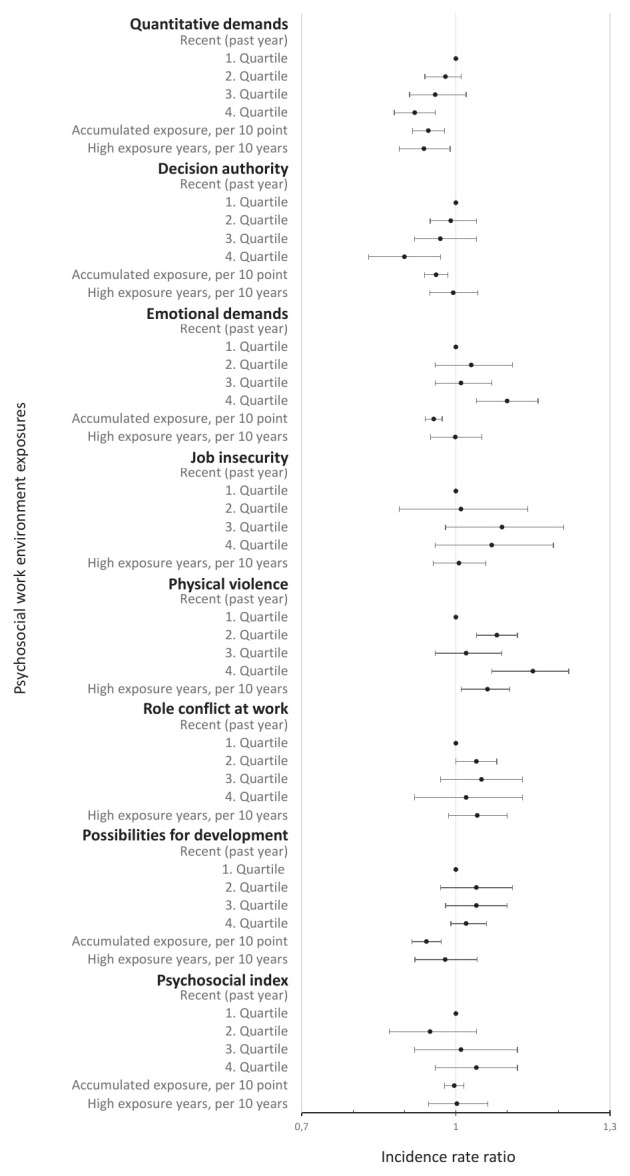
The incidence rate ratio of the psychosocial work environment exposures and all autoimmune rheumatic diseases, adjusted for model 1: age, sex, education level, calendar year, and ethnicity. The full table with results from the minimally adjusted model, model 1 and model 2 is available in supplementary appendix A3, table A2. The recent exposures all range from low (1^st^ quartile) to high (4^th^ quartile). The accumulated exposure represents the accumulated scale points of exposure across the years and is expressed per 10-scale points in the figure and per scale point in table A2. High exposure years are expressed per 10 years in the figure and per year in table A2. High exposure years includes the number of years in the highest (4^th^ quartile) of recent exposure, except for decision authority and possibilities for development at work, where the lowest quartile (1^st^ quartile) is used.

**Figure 3:**
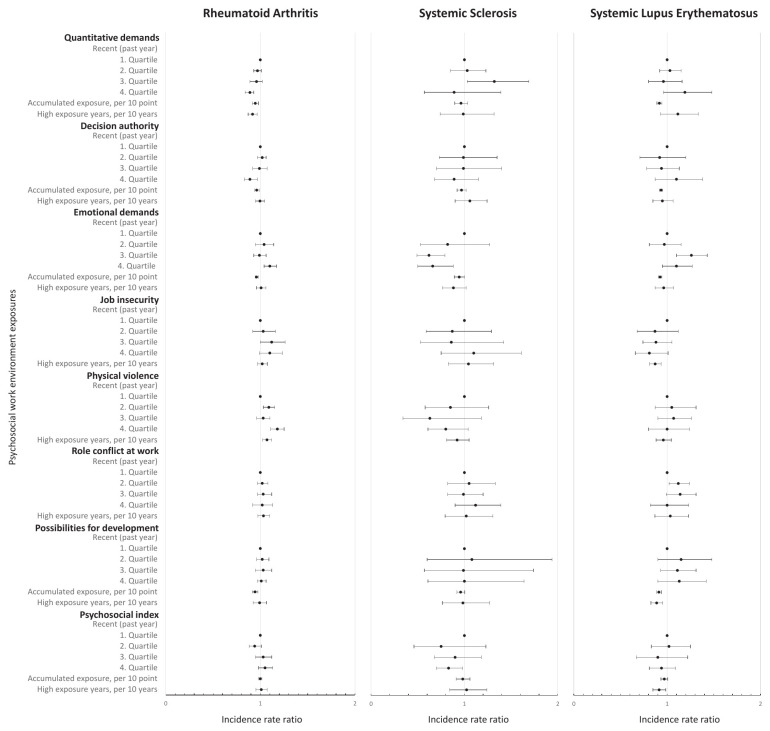
The incidence rate ratio of psychosocial work environment exposures and rheumatoid arthritis, systemic sclerosis and systemic lupus erythematosus, respectively. Adjusted for model 1: age, sex, education level, calendar year, and ethnicity. The full table with results from the minimally adjusted model, model 1 and model 2 is available in supplementary appendix A3, tables A3-A5. The recent exposures all range from low (1^st^ quartile) to high (4^th^ quartile). The accumulated exposure represents the accumulated scale points of exposure across the years and is expressed per 10-scale points in the figure and per scale point in table A2. High exposure years are expressed per 10 years in the figure and per year in table A3-A5. High exposure years includes the number of years in the highest (4th quartile) of recent exposure, except for decision authority and possibilities for development at work, where the lowest quartile (1^st^ quartile) is used.

Figures 2–3 present the association between recent, accumulated and years of high exposure for ALL and each of the psychosocial work exposures, adjusted for model 1 (main model); results from all models are available in supplementary appendix A4.

For ALL, employees in occupations with recent high, accumulated and years with high exposure to quantitative job demands had a lower risk. This repeated itself for RA, but not SS and SLE (figure 3). With additional adjustment in model 2, associations between accumulated and years of high exposure and ALL and RA disappeared (supplementary appendix A4).

In terms of decision authority, employees in occupations with recent high (4^th^ versus 1^st^ quartile: IRR 0.90, 95% CI 0.83–0.97) and accumulated (IRR 0.996, 95% CI 0.994–0.998) decision authority had lower risks of ALL. Hence, the highest quartile of decision authority during the past year had a 10% lower risk of ALL compared with the lowest quartile (model 1 in supplementary appendix A4, table A2). The same pattern was observed for RA (4^th^ versus 1^st^ quartile: IRR 0.89, 95% CI 0.82–0.97) and indicated for SS, although not statistically significantly. The lower risk between accumulated decision authority and ALL and RA disappeared in model 2 but persisted for recent high decision authority. For SLE, we observed an association with accumulated but not recent high or years with high decision authority.

Employees in occupations with recent high emotional demands had a higher risk of ALL, RA and SLE but a lower risk of SS (model 1). These associations persisted after additional adjustment in model 2. In contrast, we observed lower risks of ALL, RA, SS and SLE among employees with accumulated emotional demands, disappearing in model 2.

With regard to job insecurity, no higher risks were observed (model 1). An exception was SLE, where years with high job insecurity showed a lower risk, also indicated for recent high job insecurity.

Analyses on employees in occupations with recent and years with high risk of physical violence showed higher risks of ALL and RA, but not association with SLE or SS (models 1 and 2). For RA, the risk of disease was 18% (IRR 1.18, 95% CI 1.11–1.25) higher among employees in occupations in the highest compared to the lowest quartile of risk of violence during the past year (model 1).

For role conflicts at work, the results did not reveal an association with ALL.

The accumulated possibilities for development at work showed a lower risk of ALL (IRR 0.994, 95% CI 0.991–0.997), RA, SS and SLE (model 1), persisting in model 2 but for SLE.

For the psychosocial index, no statistically significant associations for ALL were identified. Yet, we observed a lower risk with years of high psychosocial index in the analysis of SLE and highest recent psychosocial index in the analysis of SS.

### Sensitivity analyses

Sensitivity analyses are presented in supplementary appendix A5. They generally support the main findings, except for job demands and the psychosocial index. The analyses with full follow-up since 1979 disclosed similar results as the main analyses. This was also the case when physical job strain was adjusted for, except for job demands. Here, the lowered risk of ALL after recent and years with high quantitative job demands was explained by the additional adjustment for physical job strain. The different lag time analyses also showed very similar results but for the psychosocial index. When exposure was lagged three and five years, a dose–response relationship with higher burden of recent psychosocial exposures and risk of ALL was indicated. The modified psychosocial index, excluding job demands, revealed a higher risk of ALL with a recent higher number of psychosocial work exposures (4^th^ versus 1^st^ quartile: IRR 1.19, 95% CI 1.08–1.30).

## Discussion

The presented results showed that employees in occupations with higher decision authority and, to some extent, higher possibilities for development at work, had lower risks of ALL. Additionally, employees in occupations with higher risk of physical violence were associated with higher risk of RA. Employees with higher quantitative job demands presented with a lower risk of ALL, albeit addition of physical job strain to the adjustment model somewhat explained this relationship. The results did not reveal associations for role conflicts and job insecurity. For emotional demands, the results were less clear. The main analyses did not indicate an association between the high psychosocial exposure index and ALL, but this changed with prolongation of lag time and exclusion of quantitative demands from the psychosocial index in sensitivity analyses.

Our findings corroborate the few previous studies on psychosocial work exposures and autoimmune rheumatic disease. The lower risk of autoimmune rheumatic disease with higher quantitative job demands is in line with a Swedish study on RA (1996–2003), applying both individual survey responses and JEM (26). According to the demand–control model (38), the combination of high quantitative job demands and low decision latitude can lead to high mental strain. The low decision latitude in the model includes decision authority and skill discretion, previously translated to the JEM variables of decision authority and possibility for development, used in this study (34). The combined effect of Karasek’s model was not assessed here, but we hypothesized that high quantitative job demands, low decision authority and low possibility for development per se implicate higher risk of all rheumatic diseases. The lower risk of disease with higher quantitative demands therefore contradict our expectations. The quantitative job demands JEM has previously showed contradictory results, when compared with individual questionnaire data relative to musculoskeletal pain (33). Possibly, the items for quantitative demands better capture demands in jobs with higher than lower socioeconomic position (33). This is supported by the disappearance of the lower risk when factors closely related to socioeconomic position were adjusted for, ie, family income and lifestyle factors in model 2 and physical job strain in the sensitivity analysis. As such, the association between quantitative job demands and autoimmune rheumatic disease remains unclear, and further studies, which better capture job demands, are needed to resolve the issue.

Our findings of lower risks of ALL with recent high and accumulated decision authority and possibilities for development at work are in line with our hypothesis based on the demand–control model, the Swedish study described above (26), and another Swedish population-based case–control study (1996–2015) that also indicated higher risk of RA with low decision latitude (27).

Experiencing violence can be extremely stressful, and work-related violence may increase risk of stress related disorders (39). Further, occupational trauma, including violence, is associated with higher risk of PTSD (40). As such, our findings on a high probability of violence and increased risk of RA aligns with the several studies observing increased risk of autoimmune diseases with PTSD (23–25).

The risk of RA and SLE increased with high recent exposure of emotional demands but was lower with accumulated exposure. These contradictory findings are puzzling but could also suggest a healthy worker effect. Thus, individuals with symptoms may seek jobs with less emotional demands or leave work voluntarily or involuntarily altogether.

The main analysis of the sum of the work stressors did not indicate an association, but with exclusion of quantitative job demands from the index or prolonged lag time, higher risks of rheumatic disease with higher recent burden of psychosocial stressors were indicated. Previous studies on general stressors lends some support (17, 19–22). For example, one study on perceived stress and risk of incident inflammatory arthritis, a criteria for RA (17, 18), showed association with perceived stress among individuals susceptible to developing RA (17).

### Strengths and limitations

This study builds on a pre-specified and online study protocol including a directed acyclic graph (30), minimizing risk of post-hoc decisions and selective presentation of results. Other strengths are the longitudinal study design, large study size, register-based information on jobs and outcomes, full work histories for all employees since 1979 and 22-years of follow-up time.

The application of JEM represents an additional strength, allowing the study of this register-based nationwide cohort without selective participation or attrition bias. The JEM, furthermore, enabled time-varying exposure estimation of the psychosocial work exposures for each employee based on occupational codes, which was not biased by, eg, response styles or negative affectivity. However, JEM entail risk of exposure misclassification as they estimate occupations’ average working conditions not accounting for individual exposure variability within occupational groups. This implicates a risk of non-differential misclassification of exposures (41) and hence, underestimated associations. The assumption of constant job exposures across time may have caused misclassification of exposure as exposure most probably changed over time. However, the applied JEM were sex- and age-specific, which would be expected to reduce such misclassification by taking some of the heterogeneity into account (41).

For completeness of outcome, we included both in- and outpatient records for the rheumatic diseases. Follow-up was not initiated until 1997 to reduce bias from prevalent outpatient cases, which also reduced bias from the ICD-8 to ICD-10 change in 1994. Less than 8% of the cases were registered 1979–1996 and sensitivity analyses did not indicate bias from the choice of follow-up period. To reduce risk of misclassification due to false-positive cases, we did not include the cases until their second rheumatic disease registration for the same diagnosis. Danish studies reported a positive predictive value (PPV) of 79%, when incident RA diagnoses required record ≥2 times within 90 days in rheumatology hospital departments (36). For SLE, with one SLE record and either outpatient follow-up within one year or an inpatient record due to SLE within three months, PPV was 89% (6). The median time from the first to the second registration was 42 days (interquartile range: 5–90 days), hence the two diagnoses would for the main part be registered during the same calendar year. As the majority of the cases have RA, the results of ALL are mainly driven by RA, which also enables a more precise estimation of the IRR for RA than for SS and SLE in the separate analyses.

The main analyses were adjusted based on a directed acyclic graph and time-varying covariates to reduce the risk of confounding. Yet, residual confounding can never be ruled out. In addition, we also present a minimally adjusted model and the possibly over-adjusted model 2. Model 2 included factors that were assumed mediators but could also be confounders, such as smoking and obesity. Also years of non-employment could have removed some exposure effect as employees with disease symptoms may suffer more years of unemployment. Nevertheless, model 2 largely supported the findings of the main model.

We expect our findings to be generalizable to other Nordic countries with similar labor markets and welfare systems. Further studies are needed to verify and uncover potential mechanisms to establish if these psychosocial work exposures can trigger the onset of autoimmune rheumatic disease. In addition, studies on how other potential risk factors for autoimmune rheumatic diseases, eg, crystalline silica or organic solvents (42, 43), may co-occur in specific occupations and potentially lead to additive or even synergistic effects are needed. In this perspective, it is also important to look more closely at how to include different exposures in a working life course perspective as different exposures may call for different timeframes.

### Concluding remarks

The findings of this longitudinal register-based study do not support that psychosocial work exposures are major risk factors for autoimmune rheumatic diseases in general. However, we found supporting evidence of a lower risk of autoimmune rheumatic diseases with higher decision authority and, to some extent, higher possibilities for development at work. In addition, we found support for a higher risk of RA with higher probability of physical violence. We found no supporting evidence for a higher risk of autoimmune rheumatic diseases linked to job insecurity or role conflicts at work. No clear conclusions could be drawn for quantitative job demands, emotional demands and the sum of adverse psychosocial work exposures. Thus, further studies are needed to disentangle these findings.

## Supplementary material

Supplementary material

## Data Availability

The anonymized micro data used for this study are not publicly available. Permission to access the anonymized micro data can only be granted through an affiliation with a Danish authorized environment. For additional information please visit Statistics Denmark at www.dst.dk/en/TilSalg/Forskningsservice.
