# Supplementary material for: Occupational history of psychosocial work environment exposures and risk of autoimmune rheumatic diseases – a Danish register-based cohort study
Source: Scand J Work Environ Health. 2025 Apr 27;51(3):226–36. doi: 10.5271/sjweh.4220 (PMC12071186; doi:10.5271/sjweh.4220)
Supplement: Supplementary material [file SJWEH-51-226-S001.pdf]

Occupational history of psychosocial work environment exposures and risk of autoimmune rheumatic diseases – a Danish register-based cohort study[1]

by Helena Breth Nielsen, PhD,[2] Camilla Sandal Sejbaek, PhD, Lene Wohlfahrt Dreyer, PhD, Ida EH Madsen, PhD, Esben Meulengracht Flachs, PhD, Karin Sørig Hougaard, PhD

1. Supplementary material
2. Correspondence to: Helena Breth Nielsen, National Research Centre for the Working Environment, Lersø Parkallé 105, DK-2100 Copenhagen, Denmark. [E-mail: hbn@nfa.dk]

Appendix

Occupational history of psychosocial work environment exposures and risk of autoimmune rheumatic diseases - a Danish register-based cohort study.

Appendix A1: Deviations from the protocol

Appendix A2: Flowchart of the study population

Appendix A3: Description of the JEMs and the psychosocial work environment exposure measures

Appendix A4: Results

- Results on all autoimmune rheumatic diseases (ALL, ie: rheumatoid arthritis, systemic sclerosis and systemic lupus erythematosus)
- Results on rheumatoid arthritis (RA)
- Results on systemic sclerosis (SS)
- Results on systemic lupus erythematosus (SLE)

Appendix A5: Sensitivity analyses

Appendix A1: Deviations from the protocol

Deviations from the pre-specified study protocol (1) are described below. The protocol was uploaded at Figshare prior to analyses and can be found at: https://doi.org/10.6084/m9.figshare.24745563.v1.

Covariates:

- In the protocol, it stats that all time-dependent confounders would be included at the year of the outcome (t0). In the study all time-variant confounders, except year and age, were instead included at t-1, i.e., the years before the outcome was measured to ensure they were present before the outcome.
- In the protocol highest attained education is divided in to six categories, which are reduced to five categories by combining “Master or equivalent” and “Doctoral or equivalent” into “Master or higher” in the study.
- In the protocol, smoking and obesity were supposed to be included as a measure of the recent level. Yet, we discovered that the past burden of smoking and obesity may be a better measure for potential confounding and thus included number of years with high risk of smoking or obesity instead.
- In the protocol smoking, obesity and non-employment were supposed to be included as continuous variables, but due to excessive computational run times, we had to reduce the data size. Thus, number of years with high risk of smoking and obesity as well as years of non-employment were instead categorized into: 0 years, 1-5 years, 6-10 years and more than 10 years, and included as categorical variables.

Statistics:

- We included a scale parameter to account for over-dispersion.
- Due to the few cases of SS, results from Model 2 on SS are not presented.
- All sensitivity analyses were conducted post hoc.

Minor refinements

Study population:

- In the protocol, it states that we follow employees from the year they turn 19 years old. In the study, we have explicitly stated that employees born after 2000 are excluded, as they never turn 19 years old before end of study (2018).
- In the protocol, it states that we exclude employees, who immigrate and follow employees until migration. In the study, we have refined the two criteria's to: we excluded employees, who immigrated after turning 18 years old to have the full work history, and we only follow employees until emigration.

Statistics:

- Results from Model 1 are presented in figures and the continuous measures (accumulated exposure and years with high exposure) are presented per 10 years in the figures and per year in the tables.

Reference

1. Nielsen HB, Sejbæk CS, Dreyer LW, Madsen IEH, Flachs EM, Hougaard KS. Study protocol for analyses of the association between psychosocial work environment exposures and risk of autoimmune rheumatic diseases. A study protocol from the EU-funded EXIMIOUS project. figshare. Preprint.

Appendix A2: Flowchart

Figure: Flowchart of the study population (ALL) followed from 1st January, 1997 to 31st December, 2018. N = number of individuals; PY = Person years.

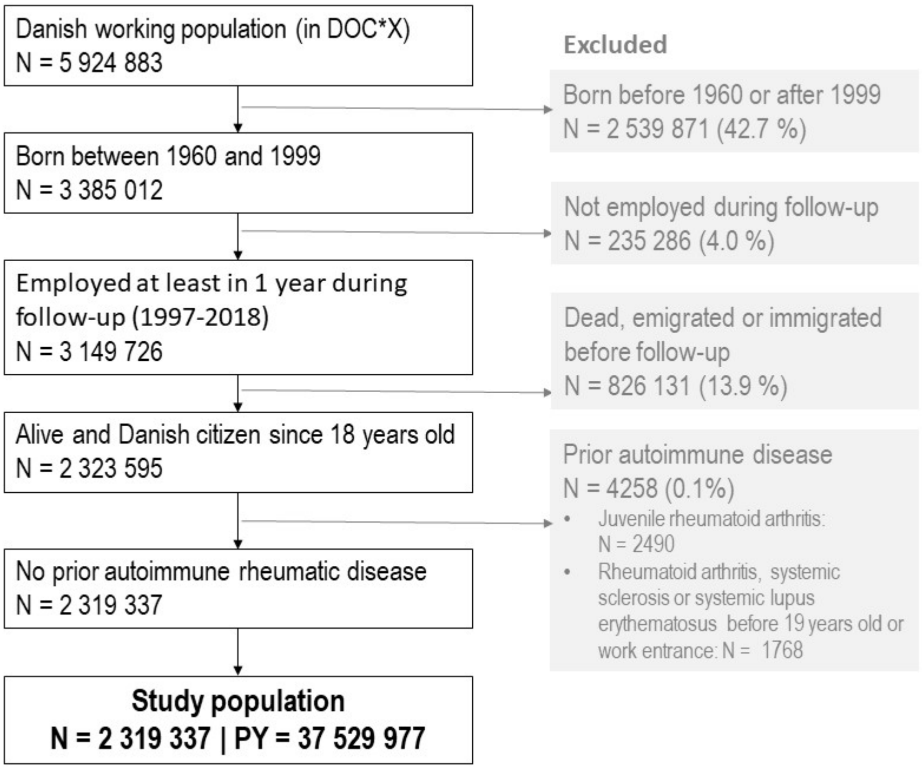

Appendix A3: Description of the JEMs

Table A1: Description of the JEMs and the psychosocial work environment exposure measures

Exposure	JEM description			Exposure measure		
	Items (self-reported)	Score	JEM estimate	**Recent exposure**	**Accumulated exposure**	**Years of high exposure**
**Quantitative demands**	1. "Do you have time enough for your work tasks?"<br>2. "Do you have deadlines that are difficult to keep?"<br>3. "Do you get unexpected work tasks that put you under time pressure?"<br>4. "Are you available outside normal working hours?"<br>5. "Do you have to work overtime?"	Mean score of the five items, which was each scored from 1 (never) to 5 (always).	Age- and sex-specific predicted mean level for each job group in 2012	Quartiles of the annual population JEM estimates at t-1.<br>1. Quartile (low) to<br>4. Quartile (high)	Sum of quantitative demand scores from entrance to the labor market and until t-1 (included)	Number of years with high quantitative demands (4. Quartile) from entrance to the labor market and until t-1 (included)
**Decision authority**	1. "Can you influence how you solve your work tasks?"<br>2. "Can you influence when you solve your work tasks?"	Mean score of the two items, which was each scored from 1 (never) to 5 (always).	Age- and sex-specific predicted mean level for each job group in 2012	Quartiles of the annual population JEM estimates at t-1<br>1. Quartile (low) to<br>4. Quartile (high)	Sum of decision authority scores from entrance to the labor market and until t-1 (included)	Number of years with low decision authority (1. Quartile) from entrance to the labor market and until t-1 (included)
**Emotional demands**	1. "Do you get emotionally involved in your work?"<br>2. "Do you have to deal with the problems of e.g. clients, patients or students in your work (not problems of your colleagues)?	Mean score of the two items, which was each scored from 1 to 5 (higher emotional demands).	Age- and sex-specific predicted mean level for each job group in 2012	Quartiles of the annual population JEM estimates at t-1.<br>1. Quartile (low) to<br>4. Quartile (high)	Sum of emotional demand scores from entrance to the labor market and until t-1 (included)	Number of years with high emotional demands (4. Quartile) from entrance to the labor market and until t-1 (included)
**Job insecurity**	1. "Are you worried about becoming unemployed?"	Dichotomized as high ("to a very high extent" or "to a high extent") and low ("to some extent", "to a low extent" or "to a very low extent")	Age- and sex-specific probability-based estimate for each job group	Quartiles of the annual population JEM probability estimates at t-1.<br>1. Quartile (low risk) to<br>4. Quartile (high risk)	-	Number of years with high risk of job insecurity (4. Quartile) from entrance to the labor market and until t-1 (included)

**Physical violence**	1.	"Have you within the past 12 months been exposed to physical violence at your workplace?"	Dichotomized as yes ("yes") or no ("no")	Age- and sex-specific probability-based estimate for each job group	Quartiles of the annual population JEM probability estimates at t.1.<br><br>1. Quartile (low risk) to<br><br>4. Quartile (high risk)	-	Number of years with high risk of physical violence (4. Quartile) from entrance to the labor market and until t-1 (included)
**Role conflicts at work**	1.	"Are contradictory demands placed on you at work?"	Dichotomized into Yes ("yes, certainly") and No ("no, not at all" or "from time to time").	Age-, sex- and period- (2000 or 2005) specific probability-based estimate for each job group	Quartiles of the annual population JEM probability estimates at t-1.<br><br>1. Quartile (low risk) to<br><br>4. Quartile (high risk)	-	Number of years with high risk of role conflicts at work (4. Quartile) from entrance to the labor market and until t-1 (included)
**Possibilities for development at work**	1.<br>2.<br>3.	"Does your work require you to take the initiative?"<br>"Do you have the possibility of learning new things through your work?"<br>"Can you use your skills or expertise in your work?"	Mean score of the three items, which was each scored from 1 (low) to 5 (higher levels of possibility for development).	Age- and sex- and period- (2000 or 2005) specific predicted mean level for each job group	Quartiles of the annual population JEM estimates at t-1.<br><br>1. Quartile (low) to<br><br>4. Quartile (high)	Sum of possibilities for development at work scores from entrance to the labor market and until t-1 (included)	Number of years with low possibilities for development at work (1. quartile) from entrance to the labor market and until t-1 (included)
**Psychosocial index**					Sum of recent adverse psychosocial exposures [A] at t-1.	Sum of adverse psychosocial exposures [A]<br><br>from entrance to the labor market and until and including recent exposure at t-1.	Number of years with high number (4. quartile) of recent adverse psychosocial exposures [A] from entrance to the labor market and until and including recent exposure at t-1.

[A] Adverse psychosocial exposures were assessed as: High quantitative demands, high emotional demands, high job insecurity, high physical violence, high role conflicts at work, low decision authority and low possibilities for development at work.

Appendix A4: Results - Analyses of all autoimmune rheumatic disease

Table A2: The IRR of quantitative demands, decision authority, emotional demands, job insecurity, physical violence, role conflicts, possibilities for development and psychosocial index on **all autoimmune rheumatic disease** (rheumatoid arthritis, systemic sclerosis and systemic lupus erythematosus)

				**Minimal adjusted model**		**Model 1 (main model)**		**Model 2**	
	PY	Cases	Cases per 10 000 PY	IRR	95%CI	IRR	95%CI	IRR	95%CI
**Quantitative demands**									
Recent (past year)									
1. Quartile (low), ref.	7 736 554	2704	3.50	1.00		1.00		1.00	
2. Quartile	7 903 307	2469	3.12	0.95	0.92-0.98	0.98	0.94-1.01	0.99	0.96-1.04
3. Quartile	7 633 075	2006	2.63	0.90	0.83-0.98	0.96	0.91-1.02	1.00	0.94-1.06
4. Quartile (high)	7 318 519	1543	2.11	0.81	0.73-0.89	0.92	0.88-0.96	0.96	0.91-1.01
Accumulated (work life)									
Per scale point	36 733 004	11067	3.01	0.999	0.992-1.005	0.995	0.991-0.998	0.999	0.995-1.003
High exposure years (work life)									
Per adverse exposure year	36 733 004	11067	3.01	0.992	0.986-0.999	0.994	0.988-0.999	1.001	0.995-1.006
**Decision authority**									
Recent (past year)									
1. Quartile (low), ref.	7 938 956	1691	2.13	1.00		1.00		1.00	
2. Quartile	7 766 608	2297	2.96	0.98	0.95-1.02	0.99	0.95-1.04	0.98	0.93-1.03
3. Quartile	7 692 795	2663	3.46	0.94	0.89-1.00	0.97	0.92-1.04	0.99	0.92-1.06
4. Quartile (high)	7 193 096	2071	2.88	0.85	0.76-0.94	0.90	0.83-0.97	0.92	0.86-0.99
Accumulated (work life)									
Per scale point	36 733 004	11067	3.01	0.999	0.995-1.004	0.996	0.994-0.998	0.999	0.996-1.002
High exposure years (work life)									
Per adverse exposure year	36 733 004	11067	3.01	1.007	0.998-1.016	1.000	0.995-1.004	1.000	0.995-1.005
**Emotional demands**									
Recent (past year)									
1. Quartile (low), ref.	7 852 292	1739	2.21	1.00		1.00		1.00	
2. Quartile	7 760 491	1667	2.15	1.02	0.93-1.11	1.03	0.96-1.11	1.05	0.99-1.12
3. Quartile	7 335 418	2303	3.14	0.97	0.91-1.02	1.01	0.96-1.07	1.04	0.99-1.08
4. Quartile (high)	7 643 254	3013	3.94	1.01	0.92-1.11	1.10	1.04-1.16	1.08	1.02-1.15
Accumulated (work life)									
Per scale point	36 733 004	11067	3.01	0.997	0.994-1.001	0.996	0.994-0.997	1.000	0.997-1.002
High exposure years (work life)									

Per adverse exposure year	36 733 004	11067	3.01	0.995	0.989-1.002	1.000	0.995-1.005	1.002	0.997-1.006
**Job insecurity**									
Recent (past year)									
1. Quartile (low risk), ref.	7 493 346	1917	2.56	1.00		1.00		1.00	
2. Quartile	7 536 601	1654	2.19	1.06	0.91-1.23	1.01	0.89-1.14	1.01	0.90-1.12
3. Quartile	7 934 888	2555	3.22	1.17	1.00-1.36	1.09	0.98-1.21	1.07	0.97-1.19
4. Quartile (high risk)	7 626 620	2596	3.40	1.18	1.01-1.38	1.07	0.96-1.19	1.02	0.91-1.15
High exposure years (work life)									
Per adverse exposure year	36 733 004	11067	3.01	1.008	1.004-1.013	1.001	0.995-1.006	1.001	0.996-1.005
**Physical violence**									
Recent (past year)									
1. Quartile (low risk), ref.	7 532 187	2184	2.90	1.00		1.00		1.00	
2. Quartile	7 910 997	1937	2.45	1.10	1.04-1.16	1.08	1.04-1.12	1.08	1.03-1.13
3. Quartile	7 384 028	1726	2.34	1.05	0.97-1.13	1.02	0.96-1.09	1.00	0.94-1.07
4. Quartile (high risk)	7 764 243	2875	3.70	1.13	1.09-1.17	1.15	1.07-1.22	1.10	1.03-1.17
High exposure years (work life)									
Per adverse exposure year	36 733 004	11067	3.01	1.004	1.001-1.007	1.006	1.001-1.010	1.006	1.001-1.010
**Role conflicts at work**									
Recent (past year)									
1. Quartile (low risk), ref.	7 698 953	3345	4.34	1.00		1.00		1.00	
2. Quartile	7 647 838	2223	2.91	1.01	0.95-1.08	1.04	1.00-1.08	1.01	0.99-1.04
3. Quartile	7 772 386	1716	2.21	1.03	0.94-1.12	1.05	0.97-1.13	1.01	0.95-1.07
4. Quartile (high risk)	8 012 400	1571	1.96	0.97	0.86-1.09	1.02	0.92-1.13	0.99	0.91-1.09
High exposure years (work life)									
Per adverse exposure year	36 788 173	11077	3.01	1.005	0.998-1.012	1.004	0.999-1.010	1.007	1.001-1.013
**Possibilities for development**									
Recent (past year)									
1. Quartile (low), ref.	7 788 223	1983	2.55	1.00		1.00		1.00	
2. Quartile	7 867 674	2310	2.94	1.01	0.93-1.09	1.04	0.97-1.11	1.05	1.00-1.11
3. Quartile	7 958 109	2381	2.99	0.98	0.89-1.07	1.04	0.98-1.10	1.05	1.00-1.10
4. Quartile (high)	7 517 571	2181	2.90	0.90	0.80-1.00	1.02	0.99-1.06	1.05	1.01-1.10
Accumulated (work life)									
Per scale point	36 788 173	11077	3.01	0.998	0.992-1.004	0.994	0.991-0.997	0.998	0.997-0.999
High exposure years (work life)									

Per adverse exposure year	36 788 173	11077	3.01	1.008	1.000-1.015	0.998	0.992-1.004	0.997	0.992-1.003
**Psychosocial index**									
Recent (past year)									
1. Quartile (low), ref.	5 651 366	1870	3.31	1.00		1.00		1.00	
2. Quartile	8 398 269	1939	2.31	0.94	0.86-1.03	0.95	0.87-1.04	0.94	0.87-1.02
3. Quartile	8 436 835	2327	2.76	1.01	0.92-1.11	1.01	0.92-1.12	0.98	0.90-1.06
4. Quartile (high)	8 871 133	2765	3.12	1.01	0.91-1.13	1.04	0.96-1.12	0.99	0.94-1.05
Accumulated (work life)									
Per scale point	36 804 358	11080	3.01	1.001	0.999-1.002	1.000	0.998-1.002	1.001	0.999-1.003
High exposure years (work life)									
Per adverse exposure year	36 804 358	11080	3.01	1.001	0.996-1.006	1.000	0.995-1.006	1.002	0.996-1.007

PY = Person years. Cases = number of individuals diagnosed with rheumatoid arthritis, systemic sclerosis or systemic lupus erythematosus. IRR = Incidence rate ration. 95%CI = 95% confidence intervals. Minimal adjusted model: adjusted for age and sex. Model 1: adjusted for age, sex, calendar year, ethnicity and highest attained education level. Model 2: adjusted for age, sex, calendar year, ethnicity, highest attained education level, years of non-employment, family income level, years of smoking and years of obesity. High exposure years: The 4th quartile in quantitative demands, emotional demands, job insecurity, physical violence and role conflicts at work, and psychosocial index, and the 1st quartile in decision authority and possibilities for development. Total number of cases and PY in ALL: 11159 cases and 37 529 977 PY. Individuals can have more than one of the three diseases, but are only included once in ALL, with their first identified case.

Appendix A4: Results - Analyses of rheumatoid arthritis

Table A3: The IRR of quantitative demands, decision authority, emotional demands, job insecurity, physical violence, role conflicts, possibilities for development and psychosocial index on **rheumatoid arthritis**

				**Minimal adjusted model**		**Model 1 (main model)**		**Model 2**	
	PY	Cases	Cases per 10 000 PY	IRR	95%CI	IRR	95%CI	IRR	95%CI
**Quantitative demands**									
Recent (past year)									
1. Quartile (low), ref.	7 738 876	2290	2.959	1.00		1.00		1.00	
2. Quartile	7 905 851	2165	2.738	0.95	0.91-0.99	0.97	0.93-1.01	0.99	0.94-1.04
3. Quartile	7 635 207	1773	2.322	0.89	0.82-0.97	0.96	0.89-1.02	0.99	0.93-1.06
4. Quartile (high)	7 319 926	1360	1.858	0.78	0.70-0.87	0.89	0.84-0.93	0.94	0.88-0.99
Accumulated (work life)									
Per scale point	36 745 401	9608	2.615	0.999	0.992-1.005	0.995	0.991-0.998	0.999	0.995-1.003
High exposure years (work life)									
Per adverse exposure year	36 745 401	9608	2.615	0.991	0.985-0.998	0.991	0.986-0.997	0.998	0.993-1.004
**Decision authority**									
Recent (past year)									
1. Quartile (low), ref.	7 940 141	1409	1.775	1.00		1.00		1.00	
2. Quartile	7 768 689	2025	2.607	1.01	0.95-1.06	1.02	0.97-1.06	1.00	0.95-1.06
3. Quartile	7 695 604	2346	3.048	0.96	0.89-1.05	0.99	0.92-1.07	1.00	0.92-1.09
4. Quartile (high)	7 195 426	1808	2.513	0.85	0.75-0.95	0.89	0.82-0.97	0.92	0.84-1.00
Accumulated (work life)									
Per scale point	36 745 401	9608	2.615	0.999	0.995-1.004	0.996	0.994-0.999	0.999	0.996-1.002
High exposure years (work life)									
Per adverse exposure year	36 745 401	9608	2.615	1.006	0.997-1.016	1.000	0.995-1.004	1.000	0.995-1.005
**Emotional demands**									
Recent (past year)									
1. Quartile (low), ref.	7 853 655	1535	1.955	1.00		1.00		1.00	
2. Quartile	7 761 656	1477	1.903	1.03	0.93-1.14	1.04	0.95-1.14	1.06	0.98-1.15
3. Quartile	7 338 140	1967	2.681	0.95	0.88-1.02	0.99	0.93-1.06	1.02	0.96-1.08
4. Quartile (high)	7 646 409	2609	3.412	1.02	0.92-1.13	1.10	1.04-1.17	1.09	1.02-1.17
Accumulated (work life)									
Per scale point	36 745 401	9608	2.615	0.998	0.995-1.001	0.996	0.994-0.998	1.000	0.997-1.003
High exposure years (work life)									

Per adverse exposure year	36 745 401	9608	2.615	0.996	0.990-1.003	1.001	0.996-1.006	1.002	0.998-1.007
**Job insecurity**									
Recent (past year)									
1. Quartile (low risk), ref.	7 495 444	1641	2.189	1.00		1.00		1.00	
2. Quartile	7 538 515	1446	1.918	1.08	0.94-1.24	1.03	0.92-1.16	1.03	0.93-1.14
3. Quartile	7 937 274	2226	2.804	1.20	1.02-1.42	1.12	1.00-1.26	1.11	0.99-1.24
4. Quartile (high risk)	7 628 627	2275	2.982	1.22	1.03-1.43	1.10	0.99-1.23	1.05	0.93-1.19
High exposure years (work life)									
Per adverse exposure year	36 745 401	9608	2.615	1.011	1.006-1.015	1.002	0.997-1.007	1.002	0.998-1.007
**Physical violence**									
Recent (past year)									
1. Quartile (low risk), ref.	7 534 257	1920	2.548	1.00		1.00		1.00	
2. Quartile	7 912 952	1665	2.104	1.11	1.03-1.19	1.09	1.03-1.15	1.09	1.03-1.15
3. Quartile	7 385 683	1496	2.026	1.06	0.96-1.15	1.03	0.96-1.10	1.00	0.94-1.07
4. Quartile (high risk)	7 766 968	2507	3.228	1.15	1.11-1.20	1.18	1.11-1.25	1.13	1.07-1.19
High exposure years (work life)									
Per adverse exposure year	36 745 401	9608	2.615	1.006	1.003-1.009	1.007	1.002-1.011	1.007	1.003-1.011
**Role conflicts at work**									
Recent (past year)									
1. Quartile (low risk), ref.	7 702 463	2894	3.757	1.00		1.00		1.00	
2. Quartile	7 650 036	1916	2.505	0.99	0.92-1.07	1.02	0.97-1.08	1.00	0.97-1.04
3. Quartile	7 773 660	1502	1.932	1.01	0.91-1.11	1.03	0.95-1.12	1.00	0.94-1.06
4. Quartile (high risk)	8 013 901	1392	1.737	0.97	0.86-1.09	1.02	0.92-1.13	1.00	0.91-1.09
High exposure years (work life)									
Per adverse exposure year	36 800 570	9619	2.614	1.005	0.998-1.012	1.003	0.997-1.009	1.006	1.000-1.013
**Possibilities for development**									
Recent (past year)									
1. Quartile (low), ref.	7 789 780	1720	2.208	1.00		1.00		1.00	
2. Quartile	7 869 977	2003	2.545	0.99	0.92-1.07	1.02	0.96-1.09	1.04	1.00-1.09
3. Quartile	7 960 525	2088	2.623	0.97	0.86-1.11	1.03	0.95-1.12	1.05	0.99-1.11
4. Quartile (high)	7 519 778	1893	2.517	0.89	0.8-0.98	1.01	0.97-1.06	1.04	1.00-1.08
Accumulated (work life)									
Per scale point	36 800 570	9619	2.614	0.998	0.992-1.004	0.994	0.991-0.997	0.998	0.997-0.999
High exposure years (work life)									
Per adverse exposure year	36 800 570	9619	2.614	1.009	1.001-1.018	0.999	0.992-1.006	0.998	0.992-1.005

**Psychosocial index**									
Recent (past year)									
1. Quartile (low), ref.	5 653 473	1626	2.876	1.00		1.00		1.00	
2. Quartile	8 400 134	1683	2.004	0.93	0.86-1.00	0.94	0.88-1.01	0.93	0.87-0.99
3. Quartile	8 438 816	2047	2.426	1.03	0.95-1.12	1.03	0.95-1.12	0.99	0.93-1.05
4. Quartile (high)	8 873 704	2390	2.693	1.02	0.92-1.14	1.05	0.98-1.13	1.00	0.95-1.06
Accumulated (work life)									
Per scale point	36 816 761	9619	2.613	1.001	0.999-1.003	1.000	0.998-1.002	1.001	0.999-1.003
High exposure years (work life)									
Per adverse exposure year	36 816 761	9619	2.613	1.002	0.997-1.008	1.001	0.995-1.007	1.002	0.996-1.008

PY = Person years. Cases = number of individuals diagnosed with rheumatoid arthritis. IRR = Incidence rate ration. 95%CI = 95% confidence intervals. Minimal adjusted model: adjusted for age and sex. Model 1: adjusted for age, sex, calendar year, ethnicity, and highest attained education level. Model 2: adjusted for age, sex, calendar year, ethnicity, highest attained education level, years of non-employment, family income level, years of smoking and years of obesity. High exposure years: The 4th quartile in quantitative demands, emotional demands, job insecurity, physical violence and role conflicts at work, and psychosocial index, and the 1st quartile in decision authority and possibilities for development. Total number of cases with RA: 9681 and PY: 37 532 750.

Appendix A4: Results - Analyses of systemic sclerosis

Note: Model 2 results are not presented due to too low number of cases

Table A4: The IRR of quantitative demands, decision authority, emotional demands, job insecurity, physical violence, role conflicts, possibilities for development and psychosocial index on **systemic sclerosis**

				**Minimal adjusted model**		**Model 1 (main model)**		**Model 2**	
	PY	Cases	Cases per 10 000 PY	IRR	95%CI	IRR	95%CI	IRR	95%CI
**Quantitative demands**									
Recent (past year)									
1. Quartile (low), ref.	7 754 880	51	0.066	1.00		1.00			
2. Quartile	7 922 508	46	0.058	1.00	0.84-1.20	1.03	0.85-1.23		
3. Quartile	7 649 686	47	0.061	1.22	0.94-1.57	1.32	1.03-1.69		
4. Quartile (high)	7 331 425	25	0.034	0.80	0.48-1.35	0.89	0.57-1.39		
Accumulated (work life)									
Per scale point	36 826 567	234	0.064	0.997	0.991-1.003	0.995	0.988-1.001		
High exposure years (work life)									
Per adverse exposure year	36 826 567	234	0.064	0.990	0.965-1.016	0.999	0.970-1.029		
**Decision authority**									
Recent (past year)									
1. Quartile (low), ref.	7 948 456	34	0.043	1.00		1.00			
2. Quartile	7 783 638	45	0.058	0.97	0.69-1.35	0.99	0.73-1.35		
3. Quartile	7 714 987	52	0.067	0.91	0.64-1.29	0.99	0.70-1.40		
4. Quartile (high)	7 211 418	38	0.053	0.80	0.63-1.00	0.89	0.68-1.15		
Accumulated (work life)									
Per scale point	36 826 567	234	0.064	0.998	0.994-1.002	0.996	0.991-1.000		
High exposure years (work life)									
Per adverse exposure year	36 826 567	234	0.064	1.017	1.002-1.032	1.006	0.992-1.021		
**Emotional demands**									
Recent (past year)									
1. Quartile (low), ref.	7 864 552	42	0.053	1.00		1.00			
2. Quartile	7 772 085	34	0.044	0.83	0.56-1.23	0.82	0.53-1.27		
3. Quartile	7 355 119	39	0.053	0.62	0.51-0.76	0.62	0.49-0.79		

4. Quartile (high)	7 666 743	54	0.070	0.64	0.53-.078	0.66	0.50-0.88
Accumulated (work life)							
Per scale point	36 826 567	234	0.064	0.995	0.990-1.000	0.993	0.987-0.999
High exposure years (work life)							
Per adverse exposure year	36 826 567	234	0.064	0.981	0.962-1.000	0.986	0.969-1.003
**Job insecurity**							
Recent (past year)							
1. Quartile (low risk), ref.	7 509 951	40	0.053	1.00		1.00	
2. Quartile	7 550 479	30	0.040	0.93	0.67-1.29	0.87	0.59-1.29
3. Quartile	7 953 804	44	0.055	0.96	0.58-1.60	0.86	0.53-1.42
4. Quartile (high risk)	7 644 265	55	0.072	1.19	0.83-1.71	1.10	0.75-1.61
High exposure years (work life)							
Per adverse exposure year	36 826 567	234	0.064	1.012	0.993-1.031	1.004	0.981-1.027
**Physical violence**							
Recent (past year)							
1. Quartile (low risk), ref.	7 550 071	52	0.069	1.00		1.00	
2. Quartile	7 924 915	39	0.049	0.90	0.61-1.34	0.85	0.58-1.26
3. Quartile	7 397 430	26	0.035	0.65	0.36-1.18	0.63	0.34-1.18
4. Quartile (high risk)	7 786 083	52	0.067	0.81	0.63-1.03	0.80	0.61-1.04
High exposure years (work life)							
Per adverse exposure year	36 826 567	234	0.064	0.991	0.981-1.001	0.993	0.981-1.006
**Role conflicts at work**							
Recent (past year)							
1. Quartile (low risk), ref.	7 725 907	68	0.088	1.00		1.00	
2. Quartile	7 664 637	44	0.057	1.02	0.80-1.32	1.05	0.82-1.33
3. Quartile	7 784 511	31	0.040	0.98	0.79-1.21	0.99	0.82-1.20
4. Quartile (high risk)	8 024 292	32	0.040	1.05	0.87-1.26	1.12	0.90-1.39
High exposure years (work life)							
Per adverse exposure year	36 881 828	234	0.063	0.996	0.976-1.017	1.002	0.977-1.027
**Possibilities for development**							
Recent (past year)							
1. Quartile (low), ref.	7 801 847	39	0.050	1.00		1.00	
2. Quartile	7 885 401	48	0.061	1.07	0.62-1.84	1.08	0.60-1.94
3. Quartile	7 976 457	46	0.058	0.97	0.57-1.63	0.99	0.57-1.74
4. Quartile (high)	7 535 642	42	0.056	0.89	0.56-1.41	1.00	0.61-1.64

Accumulated (work life)							
Per scale point	36 881 828	234	0.063	0.997	0.992-1.001	0.995	0.990-0.999
High exposure years (work life)							
Per adverse exposure year	36 881 828	234	0.063	1.012	0.994-1.030	0.998	0.976-1.021
**Psychosocial index**							
Recent (past year)							
1. Quartile (low), ref.	5 666 662	42	0.074	1.00		1.00	
2. Quartile	8 414 292	35	0.042	0.76	0.48-1.22	0.75	0.46-1.23
3. Quartile	8 454 043	47	0.056	0.90	0.71-1.13	0.90	0.68-1.18
4. Quartile (high)	8 890 797	51	0.057	0.81	0.68-0.98	0.83	0.70-0.98
Accumulated (work life)							
Per scale point	36 898 019	234	0.063	0.997	0.991-1.004	0.997	0.989-1.006
High exposure years (work life)							
Per adverse exposure year	36 898 019	234	0.063	0.995	0.978-1.013	0.997	0.978-1.017

PY = Person years. Cases = number of individuals diagnosed systemic sclerosis. IRR = Incidence rate ration. 95%CI = 95% confidence intervals. Minimal adjusted model: adjusted for age and sex. Model 1: adjusted for age, sex, calendar year, ethnicity and highest attained education level. Results from Model 2 are not presented due to the low number of cases. High exposure years: The 4th quartile in quantitative demands, emotional demands, job insecurity, physical violence and role conflicts at work, and psychosocial index, and the 1st quartile in decision authority and possibilities for development. Total number of cases with SS: 239 and PY: 37 623 900.

Appendix A4: Results - Analyses of systemic lupus erythematosus

Table A5: The IRR of quantitative demands, decision authority, emotional demands, job insecurity, physical violence, role conflicts, possibilities for development and psychosocial index of **systemic lupus erythematosus**

	PY	Cases	Cases per 10 000 PY	**Minimal adjusted model** IRR	95%CI	**Model 1 (main model)** IRR	95%CI	**Model 2** IRR	95%CI
**Quantitative demands**									
Recent (past year)									
1. Quartile (low), ref.	7 752 979	394	0.51	1.00		1.00		1.00	
2. Quartile	7 920 349	290	0.37	1.00	0.89-1.13	1.03	0.92-1.15	1.04	0.94-1.15
3. Quartile	7 648 016	208	0.27	0.91	0.73-1.15	0.96	0.80-1.16	0.99	0.81-1.21
4. Quartile (high)	7 330 283	173	0.24	1.07	0.91-1.26	1.19	0.96-1.48	1.23	0.98-1.53
Accumulated (work life)									
Per scale point	36 816 326	1386	0.38	0.993	0.989-0.997	0.991	0.987-0.994	0.999	0.992-1.005
High exposure years (work life)									
Per adverse exposure year	36 816 326	1386	0.38	0.999	0.986-1.012	1.008	0.988-1.028	1.023	1.003-1.043
**Decision authority**									
Recent (past year)									
1. Quartile (low), ref.	7 947 503	264	0.33	1.00		1.00		1.00	
2. Quartile	7 781 990	259	0.33	0.90	0.68-1.19	0.92	0.71-1.20	0.93	0.71-1.20
3. Quartile	7 712 636	291	0.38	0.88	0.72-1.08	0.94	0.78-1.13	0.97	0.80-1.17
4. Quartile (high)	7 209 498	251	0.35	0.99	0.78-1.26	1.10	0.87-1.38	1.13	0.91-1.33
Accumulated (work life)									
Per scale point	36 816 326	1386	0.38	0.995	0.992-0.999	0.993	0.990-1.000	0.998	0.994-1.001
High exposure years (work life)									
Per adverse exposure year	36 816 326	1386	0.38	1.006	0.992-1.019	0.994	0.981-1.007	0.998	0.984-1.013
**Emotional demands**									
Recent (past year)									
1. Quartile (low), ref.	7 863 647	177	0.23	1.00		1.00		1.00	
2. Quartile	7 771 133	173	0.22	0.95	0.78-1.15	0.97	0.81-1.15	0.98	0.82-1.17
3. Quartile	7 352 643	328	0.45	1.20	1.07-1.35	1.26	1.10-1.43	1.28	1.11-1.48
4. Quartile (high)	7 664 204	387	0.50	1.05	0.90-1.22	1.10	0.95-1.27	1.08	0.95-1.24
Accumulated (work life)									
Per scale point	36 816 326	1386	0.38	0.994	0.991-0.997	0.992	0.990-0.994	0.999	0.994-1.003
High exposure years (work life)									

Per adverse exposure year	36 816 326	1386	0.38	0.991	0.982-1.001	0.996	0.985-1.008	1.000	0.989-1.011
**Job insecurity**									
Recent (past year)									
1. Quartile (low risk), ref.	7 508 195	261	0.35	1.00		1.00		1.00	
2. Quartile	7 548 875	195	0.26	0.93	0.70-1.22	0.87	0.68-1.12	0.87	0.70-1.08
3. Quartile	7 951 747	318	0.40	0.95	0.80-1.14	0.88	0.74-1.05	0.86	0.73-1.01
4. Quartile (high risk)	7 642 810	291	0.38	0.89	0.75-1.06	0.81	0.66-1.01	0.76	0.60-0.94
High exposure years (work life)									
Per adverse exposure year	36 816 326	1386	0.38	0.993	0.983-1.003	0.984	0.976-0.992	0.985	0.975-0.996
**Physical violence**									
Recent (past year)									
1. Quartile (low risk), ref.	7 548 443	229	0.30	1.00		1.00		1.00	
2. Quartile	7 923 277	264	0.33	1.08	0.88-1.33	1.05	0.87-1.31	1.04	0.83-1.31
3. Quartile	7 395 938	224	0.30	1.06	0.91-1.24	1.07	0.90-1.26	1.04	0.88-1.24
4. Quartile (high risk)	7 783 969	348	0.45	1.00	0.81-1.24	1.00	0.80-1.24	0.96	0.78-1.19
High exposure years (work life)									
Per adverse exposure year	36 816 326	1386	0.38	0.996	0.989-1.004	0.997	0.989-1.006	0.999	0.990-1.007
**Role conflicts at work**									
Recent (past year)									
1. Quartile (low risk), ref.	7 722 995	424	0.55	1.00		1.00		1.00	
2. Quartile	7 662 778	290	0.38	1.13	1.03-1.24	1.12	1.02-1.24	1.12	0.99-1.26
3. Quartile	7 783 536	196	0.25	1.13	0.99-1.30	1.14	0.99-1.31	1.12	0.97-1.29
4. Quartile (high risk)	8 023 117	169	0.21	0.99	0.78-1.25	1.00	0.82-1.23	1.00	0.82-1.21
High exposure years (work life)									
Per adverse exposure year	36 871 587	1386	0.38	0.998	0.987-1.010	1.003	0.987-1.019	1.009	0.993-1.025
**Possibilities for development**									
Recent (past year)									
1. Quartile (low), ref.	7 800 551	244	0.31	1.00		1.00		1.00	
2. Quartile	7 883 559	287	0.36	1.12	0.87-1.45	1.15	0.90-1.48	1.16	0.91-1.47
3. Quartile	7 974 552	277	0.35	1.06	0.91-1.23	1.11	0.93-1.31	1.12	0.92-1.36
4. Quartile (high)	7 533 764	271	0.36	1.02	0.81-1.27	1.13	0.90-1.42	1.16	0.92-1.47
Accumulated (work life)									
Per scale point	36 871 587	1385	0.38	0.993	0.989-0.997	0.990	0.987-0.993	0.997	0.993-1.001
High exposure years (work life)									

Per adverse exposure year	36 871 587	1385	0.38	1.001	0.990-1.013	0.987	0.979-0.995	0.990	0.979-1.001
**Psychosocial index**									
Recent (past year)									
1. Quartile (low), ref.	5 664 887	230	0.41	1.00		1.00		1.00	
2. Quartile	8 412 733	237	0.28	1.02	0.81-1.27	1.02	0.83-1.25	1.01	0.82-1.24
3. Quartile	8 452 422	261	0.31	0.91	0.69-1.21	0.90	0.67-1.22	0.87	0.65-1.18
4. Quartile (high)	8 888 794	356	0.40	0.94	0.80-1.09	0.94	0.81-1.09	0.90	0.78-1.05
Accumulated (work life)									
Per scale point	36 887 772	1388	0.38	0.997	0.994-1.000	0.997	0.993-1.000	0.999	0.995-1.004
High exposure years (work life)									
Per adverse exposure year	36 887 772	1388	0.38	0.991	0.984-0.998	0.992	0.984-0.999	0.997	0.987-1.006

PY = Person years. Cases = number of individuals diagnosed with systemic lupus erythematosus. IRR = Incidence rate ration. 95%CI = 95% confidence intervals. Minimal adjusted model: adjusted for age and sex. Model 1: adjusted for age, sex, calendar year, ethnicity, and highest attained education level. Model 2: adjusted for age, sex, calendar year, ethnicity, highest attained education level, years of non-employment, family income level, years of smoking and years of obesity. High exposure years: The 4th quartile in quantitative demands, emotional demands, job insecurity, physical violence and role conflicts at work, and psychosocial index, and the 1st quartile in decision authority and possibilities for development. Total number of cases with SLE: 1400 and PY: 37 613 620.

Appendix A5: Sensitivity analyses

Table A6: Sensitivity analyses of three-year lag time, five-year lag time, follow-up since 1979 and adjusting for physical job strain. The IRR of quantitative demands, decision authority, emotional demands, job insecurity, physical violence, role conflicts, possibilities for development and psychosocial index on all autoimmune rheumatic disease (rheumatoid arthritis, systemic sclerosis and systemic lupus erythematosus), adjusted for model 1.

	**Three-years lag time**		**Five-years lag time**		**Follow-up since 1979**		**Adjusting for physical job strain**	
Model 1	IRR	95%CI	IRR	95%CI	IRR	95%CI	IRR	95%CI
**Quantitative demands**								
Recent (past year)								
1. Quartile (low), ref.	1.00		1.00		1.00		1.00	
2. Quartile	0.98	0.94-1.01	0.97	0.94-1.01	0.98	0.95-1.02	1.00	0.95-1.04
3. Quartile	0.96	0.91-1.02	0.92	0.88-0.95	0.96	0.91-1.02	1.00	0.94-1.06
4. Quartile (high)	0.92	0.88-0.96	0.91	0.84-0.99	0.93	0.88-0.98	0.98	0.92-1.04
Accumulated (work life)								
Per scale point	0.995	0.991-0.998	0.995	0.992-0.999	0.994	0.991-0.998	0.995	0.992-0.998
High exposure years (work life)								
Per adverse exposure year	0.994	0.988-0.999	0.994	0.990-0.999	0.994	0.989-1.000	0.996	0.992-1.001
**Decision authority**								
Recent (past year)								
1. Quartile (low), ref.	1.00		1.00		1.00		1.00	
2. Quartile	1.02	0.97-1.06	1.01	0.96-1.07	1.01	0.97-1.06	1.00	0.95-1.04
3. Quartile	0.93	0.87-1.00	0.95	0.89-1.00	0.99	0.93-1.05	0.99	0.92-1.07
4. Quartile (high)	0.88	0.81-0.96	0.87	0.81-0.94	0.93	0.84-1.02	0.92	0.85-1.00
Accumulated (work life)								
Per scale point	0.997	0.994-0.999	0.997	0.994-0.999	0.996	0.994-0.998	0.996	0.994-0.998
High exposure years (work life)								
Per adverse exposure year	1.000	0.994-1.005	0.999	0.994-1.004	0.999	0.995-1.003	0.998	0.993-1.002
**Emotional demands**								
Recent (past year)								
1. Quartile (low), ref.	1.00		1.00		1.00		1.00	
2. Quartile	0.99	0.89-1.10	0.99	0.90-1.10	1.02	0.96-1.09	1.05	0.97-1.13
3. Quartile	0.97	0.92-1.02	0.99	0.92-1.06	1.02	0.98-1.07	1.07	0.98-1.17
4. Quartile (high)	1.09	1.01-1.17	1.09	1.00-1.19	1.09	1.04-1.15	1.12	1.04-1.21
Accumulated (work life)								
Per scale point	0.996	0.994-0.998	0.997	0.995-0.999	0.995	0.994-0.997	0.995	0.994-0.997

High exposure years (work life)								
Per adverse exposure year	1.000	0.993-1.007	1.000	0.993-1.007	1.000	0.994-1.005	0.998	0.993-1.002
**Job insecurity**								
Recent (past year)								
1. Quartile (low risk), ref.	1.00		1.00		1.00		1.00	
2. Quartile	1.02	0.87-1.18	1.02	0.89-1.16	1.02	0.90-1.15	1.01	0.91-1.12
3. Quartile	1.07	0.92-1.24	1.05	0.95-1.17	1.09	0.98-1.20	1.10	0.99-1.22
4. Quartile (high risk)	1.10	0.96-1.25	1.11	1.02-1.21	1.06	0.95-1.18	1.05	0.93-1.19
High exposure years (work life)								
Per adverse exposure year	1.002	0.997-1.006	1.002	0.997-1.007	1.000	0.995-1.005	0.998	0.994-1.003
**Physical violence**								
Recent (past year)								
1. Quartile (low risk), ref.	1.00		1.00		1.00		1.00	
2. Quartile	1.03	0.96-1.10	1.07	1.01-1.14	1.06	1.03-1.10	1.09	1.04-1.13
3. Quartile	1.02	0.95-1.08	1.06	0.99-1.13	1.01	0.94-1.09	1.00	0.95-1.06
4. Quartile (high risk)	1.13	1.04-1.22	1.17	1.09-1.26	1.12	1.04-1.21	1.14	1.09-1.20
High exposure years (work life)								
Per adverse exposure year	1.006	1.001-1.011	1.007	1.001-1.012	1.005	1.000-1.010	1.004	1.000-1.008
**Role conflicts at work**								
Recent (past year)								
1. Quartile (low risk), ref.	1.00		1.00		1.00		1.00	
2. Quartile	1.04	1.01-1.08	1.04	0.98-1.11	1.04	1.00-1.08	1.02	0.99-1.05
3. Quartile	1.07	1.02-1.14	1.12	1.04-1.20	1.04	0.96-1.13	1.02	0.98-1.07
4. Quartile (high risk)	1.00	0.84-1.20	1.03	0.91-1.16	1.01	0.91-1.12	0.98	0.91-1.06
High exposure years (work life)								
Per adverse exposure year	1.004	0.997-1.012	1.006	1.000-1.013	1.004	0.999-1.010	1.002	0.997-1.007
**Possibilities for development**								
Recent (past year)								
1. Quartile (low), ref.	1.00		1.00		1.00		1.00	
2. Quartile	1.00	0.95-1.06	0.96	0.90-1.03	1.06	1.00-1.12	1.08	1.01-1.15
3. Quartile	1.02	0.97-1.08	1.00	0.91-1.10	1.04	0.97-1.10	1.06	0.99-1.13
4. Quartile (high)	0.98	0.91-1.06	0.98	0.89-1.08	1.03	0.99-1.07	1.05	0.99-1.12
Accumulated (work life)								
Per scale point	0.995	0.992-0.997	0.995	0.992-0.998	0.994	0.991-0.997	0.994	0.991-0.997
High exposure years (work life)								

Per adverse exposure year	1.000	0.993-1.006	0.999	0.990-1.008	0.997	0.991-1.003	0.996	0.990-1.001
**Psychosocial index**								
Recent (past year)								
1. Quartile (low), ref.	1.00		1.00		1.00		1.00	
2. Quartile	1.01	0.94-1.08	1.01	0.90-1.12	0.95	0.89-1.03	0.94	0.86-1.02
3. Quartile	1.09	1.00-1.19	1.09	0.97-1.23	1.01	0.91-1.11	0.97	0.88-1.07
4. Quartile (high)	1.10	1.02-1.19	1.11	1.01-1.22	1.02	0.94-1.10	0.99	0.92-1.07
Accumulated (work life)								
Per scale point	1.000	0.998-1.002	1.000	0.998-1.002	0.999	0.998-1.001	0.999	0.997-1.001
High exposure years (work life)								
Per adverse exposure year	1.001	0.994-1.007	1.001	0.995-1.007	0.999	0.993-1.005	0.998	0.993-1.003

IRR = Incidence rate ration. 95%CI = 95% confidence intervals. Model 1: adjusted for age, sex, calendar year, ethnicity, and highest attained education level. High exposure years: The 4th quartile in quantitative demands, emotional demands, job insecurity, physical violence and role conflicts at work, and psychosocial index, and the 1st quartile in decision authority and possibilities for development.

Table A7: Sensitivity analyses including a modified psychosocial index. The IRR of the modified psychosocial index and risk of all autoimmune rheumatic disease (rheumatoid arthritis, systemic sclerosis and systemic lupus erythematosus), adjusted as in Model 1.

Model 1	**IRR**	**95%CI**
**Modified psychosocial index**[A]		
Recent (past year)		
1. Quartile (low), ref.	1.00	
2. Quartile	1.09	1.01-1.17
3. Quartile	1.07	1.03-1.12
4. Quartile (high)	1.19	1.08-1.30
Accumulated (work life)		
Per scale point	1.000	0.998-1.002
High exposure years (work life)		
Per adverse exposure year	1.000	0.998-1.002

[A]: The modified psychosocial index included: emotional demands, job insecurity, physical violence, role conflicts at work, decision authority and possibilities for development at work. IRR = Incidence rate ration. 95%CI = 95% confidence intervals. Model 1: adjusted for age, sex, calendar year, ethnicity, and highest attained education level. High exposure years: The 4th quartile in the modified psychosocial index.
